# Pulmonary Embolism in Hospitalized COVID-19 Patients: Incidence, Clinical Predictors, and Short-Term Outcomes

**DOI:** 10.3390/jcm15083117

**Published:** 2026-04-19

**Authors:** Cristiana Adina Avram, Maria-Laura Craciun, Ana-Maria Pah, Stela Iurciuc, Simina Crisan, Cristina Vacarescu, Ioana Cotet, Claudia Raluca Balasa Virzob, Dan Alexandru Surducan, Claudiu Avram

**Affiliations:** 1 Department of Internal Medicine I, “Victor Babes” University of Medicine and Pharmacy Timisoara, Eftimie Murgu Square 2, 300041 Timisoara, Romania; avram.adina@umft.ro; 2 Cardiology Department, “Victor Babes” University of Medicine and Pharmacy Timisoara, Eftimie Murgu Square 2, 300041 Timisoara, Romania; laura.craciun@umft.ro (M.-L.C.); simina.crisan@umft.ro (S.C.);; 3 Department of General Medicine, Doctoral School, “Victor Babes” University of Medicine and Pharmacy Timisoara, Eftimie Murgu Square 2, 300041 Timisoara, Romania; ioana.cotet@umft.ro; 4 Department of Clinic Nursing, “Victor Babes” University of Medicine and Pharmacy Timisoara, Eftimie Murgu Square 2, 300041 Timisoara, Romania; virzob.claudia@umft.ro; 5 Department of Functional Sciences, Discipline of Public Health, Center for Translational Research and Systems Medicine, “Victor Babes” University of Medicine and Pharmacy Timisoara, Eftimie Murgu Square 2, 300041 Timisoara, Romania; 6 Centre for Translational Research and Systems Medicine, Faculty of Medicine, “Victor Babes” University of Medicine and Pharmacy Timisoara, Eftimie Murgu Square 2, 300041 Timisoara, Romania; 7 Department XVI-Balneology, Medical Recovery and Rheumatology, “Victor Babes” University of Medicine and Pharmacy Timisoara, Eftimie Murgu Square 2, 300041 Timisoara, Romania; avram.claudiu@umft.ro; 8 Transdisciplinary Research Center for Medical Rehabilitation, Balneology and Rheumatology (CTRMBR), “Victor Babes” University of Medicine and Pharmacy Timisoara, Eftimie Murgu Square 2, 300041 Timisoara, Romania

**Keywords:** COVID-19, pulmonary embolism, venous thromboembolism, D-dimer, immobilization, risk stratification, anticoagulation, long COVID

## Abstract

**Background/Objectives**: Pulmonary embolism (PE) represents a major thrombotic complication in hospitalized patients with coronavirus disease 2019 (COVID-19), yet data on its incidence, clinical predictors, and short-term outcomes in actual cohorts remain heterogeneous. **Methods**: We conducted a retrospective observational cohort study including 395 consecutive adults hospitalized with RT-PCR-confirmed COVID-19 at a tertiary infectious diseases center between March 2020 and December 2024. Clinical, laboratory, imaging, and treatment data were extracted from electronic records, and PE was defined by computed tomography pulmonary angiography. Univariable and multivariable logistic regression analyses were used to identify independent predictors of PE in the subset of patients who underwent CTPA (*n* = 120), in whom PE status was definitively ascertained (47 with PE and 73 without PE). **Results**: Pulmonary embolism was diagnosed in 47 patients (11.9%). Patients with PE more frequently had prior venous thromboembolism (19.1% vs. 8.3%) and prolonged immobilization (61.7% vs. 23.0%), and were more often admitted to the intensive care unit (12.8% vs. 4.3%) than those without PE. Peak D-dimer levels were almost ten-fold higher in the PE group (median 5322 vs. 529.5 µg/L). In multivariable logistic regression, peak D-dimer was independently associated with PE (per log-unit increase, adjusted OR 3.9, 95% CI 2.1–7.1), and prolonged immobilization conferred a substantially higher risk of PE (adjusted OR 5.1, 95% CI 2.4–10.9). Patients with PE experienced more complex hospital courses and more frequent need for advanced therapies, although in-hospital mortality did not differ significantly between groups. **Conclusions**: In hospitalized COVID-19 patients, PE is frequent and closely linked to marked D-dimer elevation and acquired in-hospital risk factors, particularly prolonged immobilization. This evidence supports the use of dynamic D-dimer assessment and careful evaluation of immobilization status to improve risk stratification, guide decisions on diagnostic imaging and anticoagulation intensity, and identify patients who may benefit from closer post-discharge cardiovascular follow-up (this hypothesis requires confirmation in future prospective studies).

## 1. Introduction

Severe acute respiratory syndrome coronavirus 2 (SARS-CoV-2) infection is now recognized as a systemic disease extending beyond the respiratory tract, with substantial cardiovascular and thrombotic complications across the spectrum of disease severity. Among these complications, venous thromboembolism (VTE), particularly pulmonary embolism (PE), has emerged as one of the most clinically significant and potentially life-threatening manifestations in hospitalized patients with coronavirus disease 2019 (COVID-19). Early reports during the pandemic demonstrated unexpectedly high rates of thrombotic complications despite standard thromboprophylaxis, suggesting a unique prothrombotic phenotype associated with COVID-19 [1,2,3,4]. Subsequent meta-analyses estimated pooled PE incidence rates of approximately 14–23% in hospitalized patients, with even higher rates among those requiring intensive care support [2,3,4].

The pathophysiology of COVID-19-associated thrombosis is multifactorial and entails complex interactions among inflammation, endothelial injury, platelet activation, and dysregulated coagulation pathways. SARS-CoV-2 infection induces endothelial dysfunction through both direct viral invasion and indirect inflammatory mechanisms, resulting in endothelial activation, reduced nitric oxide bioavailability, oxidative stress, and a procoagulant state that promotes both microvascular and macrovascular thrombosis [5,6,7]. This endotheliopathy has led to the conceptualization of COVID-19 as a vascular disease characterized by immunothrombosis and widespread endothelial injury [8].

Elevated biomarkers of coagulation and inflammation, particularly D-dimer, fibrinogen, and inflammatory cytokines, have consistently been associated with thrombotic complications and adverse outcomes in COVID-19 [9]. Elevated IL-6, cardiac biomarkers, and clinical factors have also been shown to predict unfavorable outcomes in COVID-19 sepsis cohorts, suggesting that integrated biomarker models may enhance risk stratification. In an Eastern European prospective cohort, these variables were significantly associated with mortality and adverse outcomes in hospitalized patients with COVID-19 sepsis [10]. D-dimer levels above specific thresholds have demonstrated high sensitivity for predicting PE and mortality in hospitalized patients, supporting their role as clinically useful markers for risk stratification [9]. In addition, clinical factors such as advanced age, male sex, obesity, smoking, immobilization, and pre-existing cardiovascular disease further increase thrombotic risk, indicating the convergence of infection-related and traditional VTE risk factors [4,9].

Despite rising recognition of the thrombotic burden in COVID-19, several clinically relevant gaps remain. First, the predictors of PE in hospitalized COVID-19 populations continue to vary across studies, likely reflecting heterogeneity in patient characteristics, disease severity, diagnostic strategies, and therapeutic exposures during hospitalization. Second, the relationship between acute thrombotic complications and subsequent cardiovascular risk is still incompletely understood. Identifying patients at highest risk for PE during hospitalization may afford important insights into those who could benefit from intensified monitoring, extended thromboprophylaxis, or targeted cardiovascular follow-up after discharge, thereby improving both short- and long-term outcomes [11]. Importantly, most large multicenter cohorts and meta-analyses describing PE in COVID-19 were conducted during early pandemic waves, before stabilization of vaccination coverage and evolution of institutional anticoagulation protocols. In contrast, the present single-center cohort spans 2020–2024 and reflects a mature phase of the pandemic in an Eastern European setting, where thromboprophylaxis strategies, case-mix, and health-system constraints differ from those in Western European or North American networks. These data therefore complement multicenter evidence by providing real-world observations on pulmonary embolism risk among hospitalized COVID-19 patients in an Eastern European tertiary center [2,3].

A previous analysis from our group evaluated pulmonary embolism in a smaller Romanian cohort of hospitalized COVID-19 patients, focusing primarily on prevalence, baseline risk factors, and immediate in-hospital outcomes during the early pandemic waves [12]. That study did not incorporate dynamic biomarker trajectories, detailed characterization of immobilization and anticoagulation patterns, or extended enrollment across subsequent pandemic phases. Building on these preliminary observations, the present work analyzes an expanded and updated cohort from 2020 to 2024, integrates peak biomarker values, systematically quantifies in-hospital immobilization and anticoagulation intensity, and provides a more granular description of PE localization, severity, and short-term clinical course. Importantly, the extended inclusion period (2020–2024) allowed the cohort to capture multiple phases of the pandemic, including pre-vaccination waves and later periods characterized by evolving vaccination coverage and modified thromboprophylaxis strategies. This temporal breadth provides a unique opportunity to evaluate PE risk in a more mature phase of COVID-19 care, complementing earlier studies conducted predominantly during the first pandemic waves. By doing so, this study offers context-specific data from an Eastern European tertiary center and addresses how evolving in-hospital practices and acquired risk factors shape PE risk in contemporary COVID-19 care.

## 2. Materials and Methods

### 2.1. Study Design and Population

This retrospective observational cohort study included consecutive adult patients hospitalized with confirmed coronavirus disease 2019 (COVID-19) at the “Dr. Victor Babeș” Clinical Hospital of Infectious Diseases and Pneumophthisiology, Timișoara, Romania, between March 2020 and December 2024. The study was conducted in accordance with the principles of the Declaration of Helsinki and followed the Strengthening the Reporting of Observational Studies in Epidemiology (STROBE) recommendations for cohort studies.

Eligible patients were ≥18 years of age and had SARS-CoV-2 infection confirmed by reverse transcription polymerase chain reaction (RT-PCR) from nasopharyngeal swabs. Patients transferred to or from external institutions without complete medical records or lacking essential diagnostic data required for pulmonary embolism (PE) assessment were excluded. Only the first hospitalization episode was considered for patients with multiple admissions.

The final study cohort comprised 395 patients meeting all eligibility criteria.

### 2.2. Data Collection

Clinical, laboratory, imaging, and treatment data were extracted from electronic medical records using a standardized data collection protocol. Variables included demographic characteristics (age, sex); cardiovascular risk factors (smoking status, body mass index); comorbidities, including cardiovascular disease and prior venous thromboembolism; clinical severity indicators at admission (oxygen saturation, need for oxygen therapy); hospitalization characteristics (duration of hospitalization, intensive care unit [ICU] admission, duration of ICU stay); laboratory parameters at admission and during hospitalization (complete blood count, coagulation parameters, inflammatory biomarkers, renal and hepatic function markers); anticoagulation therapy (type, dose, duration); imaging findings, including computed tomography pulmonary angiography (CTPA); and clinical outcomes, including ICU transfer, thrombolytic therapy, and in-hospital mortality.

Laboratory parameters were recorded both at admission and peak values during hospitalization when available. For biomarkers with repeated measurements, the highest recorded value during hospitalization was used for outcome analyses.

### 2.3. Definition of Pulmonary Embolism

Pulmonary embolism was defined as the presence of intraluminal filling defects consistent with thrombus on contrast-enhanced computed tomography pulmonary angiography (CTPA), interpreted by experienced radiologists. PE localization was categorized as central (main or lobar arteries) or peripheral (segmental or subsegmental branches).

Patients without radiological evidence of PE on CTPA were classified as PE-negative.

Computed tomography pulmonary angiography (CTPA) was not performed systematically in all patients, but was requested based on clinical suspicion of pulmonary embolism according to institutional clinical practice during the pandemic period. Indications for CTPA included sudden worsening of respiratory status, unexplained hypoxemia disproportionate to pulmonary imaging findings, marked elevation of D-dimer levels, clinical signs suggestive of venous thromboembolism, or hemodynamic deterioration without an alternative explanation.

Consequently, pulmonary embolism was diagnosed only in patients undergoing clinically indicated CTPA. Because systematic screening imaging was not performed in the entire cohort, the reported prevalence of pulmonary embolism should be interpreted as the proportion of confirmed cases among hospitalized patients rather than the true population incidence.

### 2.4. Outcomes

The primary outcome of the study was the occurrence of pulmonary embolism during hospitalization.

Secondary outcomes included: PE severity (central vs. peripheral localization), need for thrombolytic therapy, ICU admission related to thromboembolic complications, in-hospital mortality. For the analysis of predictors of PE, the outcome was the presence of CTPA-confirmed PE during hospitalization among patients who underwent CTPA. Patients who did not undergo CTPA were not classified as PE-negative for this analysis and were therefore excluded from the multivariable prediction model.

### 2.5. Anticoagulation Therapy

Anticoagulation regimens were recorded, including prophylactic, intermediate, and therapeutic dosing strategies, according to institutional protocols and contemporary clinical guidelines during the study period. Pharmacologic thromboprophylaxis was generally initiated within the first 24 h of hospital admission in the absence of contraindications, using standard prophylactic-dose low-molecular-weight heparin in most patients. Escalation to intermediate or therapeutic anticoagulation was considered in patients with markedly elevated or rapidly rising D-dimer levels, clinical deterioration raising suspicion of venous thromboembolism, or confirmed PE on CTPA. For the present analysis, we additionally recorded the intensity of anticoagulation prior to PE diagnosis (prophylactic vs. intermediate vs. therapeutic), timing of anticoagulation initiation in relation to admission, and differences in pre-PE anticoagulation intensity between patients who subsequently developed PE and those who did not. The duration of anticoagulation therapy during hospitalization was also documented.

### 2.6. Statistical Analysis

Continuous variables were assessed for normality using the Shapiro–Wilk test. Normally distributed variables were expressed as mean ± standard deviation (SD), while non-normally distributed variables were presented as median with interquartile range (IQR). Categorical variables were reported as counts and percentages.

Comparisons between patients with and without pulmonary embolism were performed using: Student’s *t*-test or Mann–Whitney U test for continuous variables, as appropriate, and Chi-square test or Fisher’s exact test for categorical variables.

Univariable logistic regression analysis was performed to identify potential predictors of pulmonary embolism. These regression analyses were restricted to the subset of patients who underwent CTPA (*n* = 120), in whom PE status was definitively ascertained (47 with PE and 73 without PE). In the CTPA subcohort, 47 patients experienced PE, yielding an events-per-variable ratio close to the commonly recommended threshold for multivariable logistic regression. The final multivariable model included 4 predictors (peak D-dimer, prolonged immobilization, prior venous thromboembolism, and ICU admission), and no further variables were added to avoid overfitting. Patients who did not undergo CTPA (*n* = 275) were not considered PE-negative and were excluded from these models to avoid outcome misclassification. Variables with *p* < 0.10 in univariable analysis and clinically relevant covariates were entered into a multivariable logistic regression model to identify independent predictors. Results were reported as odds ratios (ORs) with 95% confidence intervals (CIs).

Multicollinearity among covariates was assessed using variance inflation factors (VIF), with VIF < 2 considered acceptable. Model calibration was evaluated using the Hosmer–Lemeshow goodness-of-fit test, and discrimination was assessed using receiver operating characteristic (ROC) curve analysis with area under the curve (AUC) estimation in the CTPA subcohort.

In addition to the primary multivariable model including peak D-dimer, prolonged immobilization, prior venous thromboembolism, and ICU admission, we performed sensitivity analyses using separate multivariable models centered on each key predictor. Specifically, we fitted models with peak D-dimer or prolonged immobilization as the main variable of interest, adjusted for demographic and clinical covariates (age, sex, and body mass index) that were not expected to lie on the causal pathway between COVID-19 severity and PE. These sensitivity models were designed to mitigate potential collinearity among predictors and to explore the robustness of the observed associations.

For anticoagulation, we summarized the distribution of prophylactic, intermediate, and therapeutic dosing over time using descriptive statistics and did not perform formal hypothesis testing or multivariable modelling of anticoagulation intensity versus clinical outcomes. Given the limited number of PE events and the strong association between anticoagulation intensity and markers of disease severity, any adjusted comparison would have been highly susceptible to residual confounding and overfitting, so we deliberately restricted this analysis to a descriptive, hypothesis-generating approach.

Missing data were handled using complete-case analysis, given the retrospective nature of the dataset and the relatively low proportion of missing values for primary variables. For each variable included in the multivariable model, the proportion of missing values was quantified and was below 5% (peak D-dimer 2%, prolonged immobilization 0%, prior venous thromboembolism 0%, ICU admission 0%). Overall, 96% of patients in the CTPA subcohort had complete data on all variables required for the primary multivariable model and were included in the complete-case analysis. We assumed that missingness for laboratory covariates was at least missing at random conditional on observed clinical characteristics and outcomes, so that a complete-case approach would yield approximately unbiased estimates, acknowledging the potential loss of efficiency.

A two-sided *p*-value < 0.05 was considered statistically significant. Statistical analyses were performed using SPSS Statistics version 26.0 (IBM Corp., Armonk, NY, USA).

### 2.7. Ethical Approval

The study was conducted in accordance with the principles of the Declaration of Helsinki and was approved by the Ethics Committee of the “Dr. Victor Babeș” Clinical Hospital of Infectious Diseases and Pulmonology, Timișoara, Romania (approval no. 11900/16 December 2025). All patients provided written informed consent for the use of their anonymized clinical data for research purposes.

## 3. Results

### 3.1. Study Population and Prevalence of Pulmonary Embolism

A total of 395 hospitalized patients with confirmed COVID-19 were included in the final analysis. During hospitalization, 120 patients (30.4%) of the total cohort underwent computed tomography pulmonary angiography (CTPA) based on clinical suspicion of pulmonary embolism. Only these 120 patients, in whom PE status was definitively confirmed or excluded by CTPA (47 with PE and 73 without PE), were included in the logistic regression analyses of predictors of PE. The remaining 275 patients without CTPA were not classified as PE-negative and were excluded from the prediction models. Among those investigated, pulmonary embolism was confirmed in 47 patients. This corresponds to a prevalence of 39.2% among patients undergoing CTPA and 11.9% relative to the entire hospitalized cohort.

Baseline characteristics are presented for the entire cohort to describe the overall hospitalized population, whereas regression analyses evaluating predictors of pulmonary embolism were restricted to the subgroup of patients who underwent CTPA and had imaging-confirmed PE status.

The mean age of patients with pulmonary embolism was 68.7 ± 13.4 years compared with 72.1 ± 13.6 years among patients without PE. Body mass index was slightly higher in the PE group (28.4 ± 5.1 kg/m^2^ vs. 28.0 ± 5.3 kg/m^2^), although this difference was not clinically substantial.

Baseline demographic and clinical characteristics are presented in Table 1.

### 3.2. Clinical Severity and Hospital Course

Markers of disease severity were significantly more pronounced among patients with pulmonary embolism. ICU admission occurred more frequently in the PE group compared with patients without PE (12.8% vs. 4.3%). Similarly, prolonged immobilization was substantially more common among patients with PE (61.7% vs. 23.0%), indicating an important contribution of acquired thrombotic risk factors during hospitalization.

The median duration of hospitalization was comparable between groups, although patients with PE demonstrated greater clinical complexity, including higher rates of complications and need for advanced therapies.

### 3.3. Laboratory Findings

Significant differences were observed in coagulation parameters between groups.

**Figure 1:**
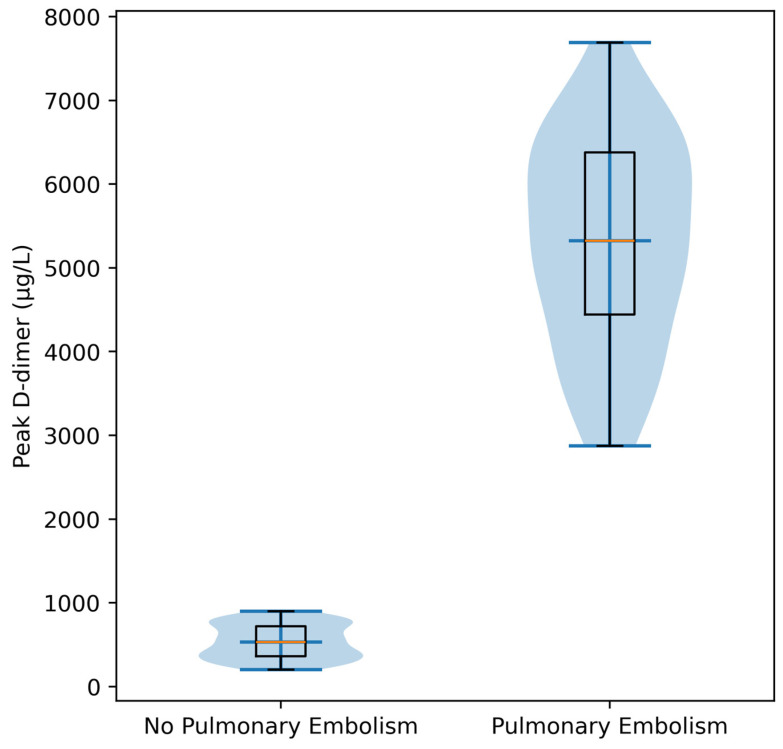
Peak D-dimer levels according to pulmonary embolism status. Patients with pulmonary embolism exhibited markedly higher peak D-dimer concentrations compared with those without pulmonary embolism, reflecting the strong association between hypercoagulability and thrombotic complications (*p* < 0.001). The violin plots represent the distribution of values (kernel density), with overlaid boxplots indicating the interquartile range (IQR), median (central line), and range (whiskers).

Patients with pulmonary embolism had markedly elevated peak D-dimer levels compared with those without PE: PE: median 5322 µg/L and No PE: median 529.5 µg/L. This marked difference in D-dimer levels between groups is illustrated in Figure 1.

This represents approximately a ten-fold difference, confirming the strong association between hypercoagulability and thrombotic complications.

Inflammatory markers were also elevated among patients with PE. Median C-reactive protein at admission was higher in the PE group (113.8 mg/L vs. 108.0 mg/L), consistent with a more pronounced inflammatory response. Interleukin-6 and fibrinogen levels showed similar trends, supporting the concept of thromboinflammation as a central mechanism.

Laboratory comparisons are summarized in Table 2.

### 3.4. Characteristics of Pulmonary Embolism

Among the 47 patients diagnosed with pulmonary embolism, thrombus localization involved both proximal and distal pulmonary arterial branches, with segmental and subsegmental involvement representing the most common patterns. A subset of patients presented with central embolism affecting main or lobar pulmonary arteries, reflecting clinically significant thrombotic burden.

Thrombolytic therapy was administered in selected severe cases, and ICU transfer directly related to PE occurred in a subset of patients. PE-related mortality was recorded but remained relatively low compared with the overall mortality of the cohort.

Detailed PE characteristics are presented in Table 3.

### 3.5. Anticoagulation Patterns and Clinical Context

Anticoagulation therapy was administered according to institutional protocols, including prophylactic and therapeutic dosing strategies. Patients with confirmed PE received therapeutic anticoagulation, with duration tailored according to clinical evolution.

The incidence of hemorrhagic complications remained low, supporting the safety of anticoagulation strategies used in this population.

#### Anticoagulation Intensity and Timing in Relation to PE

Among the overall cohort, pharmacologic thromboprophylaxis was initiated within 24 h of admission in 92.4% of patients, with a median time from hospital admission to anticoagulation start of 0.9 (IQR 0.5–1.4) days. Before PE diagnosis, most patients who subsequently developed PE were receiving prophylactic-dose anticoagulation, whereas only a minority were on intermediate or therapeutic regimens (prophylactic 80.9%, intermediate 12.8%, therapeutic 6.4%). In contrast, among patients who did not develop PE, the distribution of anticoagulation intensity at comparable time points was prophylactic 86.5%, intermediate 8.3%, and therapeutic 5.2%. The proportion of patients already on therapeutic-dose anticoagulation prior to PE diagnosis was low in both groups, suggesting that incident PE occurred predominantly despite standard prophylactic regimens rather than during full-dose anticoagulation.

Because anticoagulation strategies were strongly driven by clinical severity and dynamic deterioration, we did not perform formal statistical comparisons of anticoagulation intensity across outcome groups, and these data are presented descriptively.

### 3.6. Predictors of Pulmonary Embolism

In the CTPA subcohort (*n* = 120), univariable logistic regression analysis identified several variables associated with pulmonary embolism, including elevated D-dimer levels, prolonged immobilization, prior history of venous thromboembolism, ICU admission, and markers of disease severity. In the multivariable logistic regression model restricted to patients with CTPA-confirmed PE status, peak D-dimer remained independently associated with PE (per log-unit increase, adjusted OR 3.9, 95% CI 2.1–7.1), and prolonged immobilization conferred a substantially higher risk of PE (adjusted OR 5.1, 95% CI 2.4–10.9). ICU admission also contributed to risk stratification, but with a more modest effect size, whereas traditional cardiovascular risk factors did not retain independent associations with PE after adjustment.

**Figure 2:**
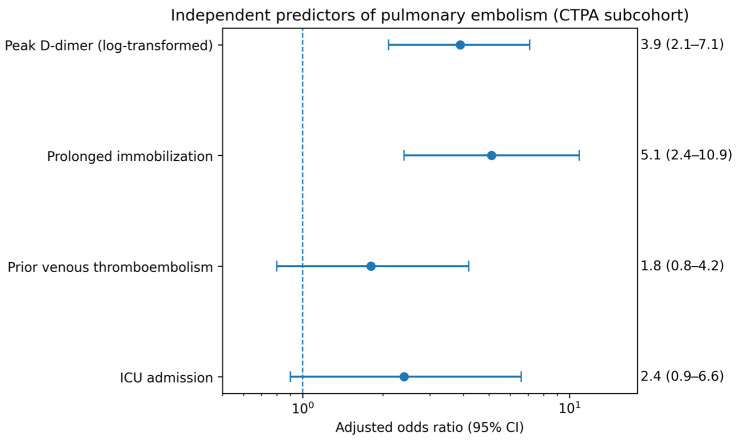
Independent predictors of pulmonary embolism in the CTPA subcohort. Forest plot showing adjusted odds ratios (ORs) with 95% confidence intervals (CIs) derived from the multivariable logistic regression model restricted to patients who underwent CTPA (*n* = 120). Points represent adjusted odds ratios and horizontal lines indicate 95% confidence intervals. Peak D-dimer (log-transformed) and prolonged immobilization were independently associated with pulmonary embolism and ICU admission showed weaker, non-significant associations. The vertical reference line represents an OR of 1.

The final model demonstrated good discrimination for predicting PE in the CTPA subcohort, with an area under the receiver operating characteristic curve (AUC) of 0.80. The complete results of the multivariable logistic regression model, including adjusted odds ratios, 95% confidence intervals, and *p*-values, are presented in Table 4, and the independent predictors are graphically depicted in Figure 2.

To further assess the potential impact of collinearity among predictors, we performed additional sensitivity analyses using separate multivariable models centered on each key predictor. When peak D-dimer was modeled together with age, sex, and body mass index, without inclusion of other PE-related clinical variables, it remained strongly associated with PE (adjusted OR 3.7, 95% CI 2.0–6.8). Similarly, prolonged immobilization preserved a robust association with PE in a model adjusted for age, sex, and body mass index (adjusted OR 4.8, 95% CI 2.2–10.3). These sensitivity analyses indicate that the observed effects of peak D-dimer and immobilization are not solely driven by collinearity with other markers of disease severity and support the stability of the primary model.

### 3.7. Clinical Outcomes

Pulmonary embolism was associated with worse clinical outcomes compared with patients without PE. ICU admission rates were higher, and patients experienced more complications during hospitalization. Although overall mortality differences did not reach statistical significance, PE represented a marker of disease severity and systemic thromboinflammatory activation.

### 3.8. Pathophysiological Implications

The strong association between elevated D-dimer levels, immobilization, and pulmonary embolism supports the concept of COVID-19-associated immunothrombosis and endothelial dysfunction as central mechanisms underlying thrombotic complications. These findings also provide mechanistic insight into the potential contribution of acute thrombotic events to persistent vascular dysfunction and long-term cardiovascular vulnerability observed in post-acute COVID-19 syndromes.

## 4. Discussion

In this retrospective cohort study of hospitalized patients with COVID-19, pulmonary embolism was diagnosed in approximately one in nine hospitalized patients undergoing computed tomography pulmonary angiography, highlighting the substantial thrombotic burden associated with SARS-CoV-2 infection. The present study identified three main observations. First, pulmonary embolism was strongly associated with markers of hypercoagulability and acquired thrombotic risk, particularly elevated D-dimer levels and prolonged immobilization. Second, thromboembolic complications were linked with more severe clinical courses, including higher rates of intensive care unit admission and heightened healthcare resource utilization. Third, the observed associations between thromboinflammatory biomarkers and pulmonary embolism provide mechanistic insight into the potential contribution of acute thrombotic injury to persistent cardiovascular vulnerability in the context of post-acute COVID-19 syndromes. In the CTPA subcohort, our multivariable logistic regression model demonstrated good discrimination for predicting PE, with an area under the receiver operating characteristic curve (AUC) of 0.80. Importantly, to avoid misclassification of patients without CTPA as PE-negative, this prediction model was restricted to the subset of patients who underwent CTPA and had imaging-confirmed PE status. Patients who did not undergo CTPA were considered to have unascertained PE status and were therefore excluded from the regression analyses, thereby reducing the risk of bias in the estimated odds ratios and model discrimination. Importantly, this study is subject to spectrum bias and verification bias, as CTPA was performed only in patients with clinical suspicion of pulmonary embolism rather than systematically in the entire cohort. Consequently, the identified predictors should be interpreted as applicable to patients with suspected PE rather than to all hospitalized COVID-19 patients. This selective imaging strategy may have enriched the CTPA subcohort for higher-risk individuals, thereby potentially inflating effect sizes and limiting generalizability. Future studies with systematic screening or prospective designs are needed to validate these predictors in unselected populations.

The incidence of pulmonary embolism observed in this cohort is consistent with prior reports describing high thrombotic rates among hospitalized COVID-19 patients, although estimates vary widely depending on disease severity and diagnostic strategies. Variability in reported pulmonary embolism incidence across studies may partly reflect differences in diagnostic strategies, particularly whether CTPA was performed systematically or only in patients with clinical suspicion of thromboembolism. Consequently, the observed prevalence should be interpreted as the proportion of confirmed PE cases among hospitalized patients rather than the true population incidence. These data are consistent with previous reports describing high rates of pulmonary embolism and strong associations with coagulation biomarkers in hospitalized COVID-19 cohorts from Eastern Europe [12]. A systematic review and meta-analysis by Suh et al. reported pooled PE rates of approximately 16.5% in hospitalized patients, with significantly higher prevalence among critically ill populations [13]. Similarly, observational studies have demonstrated that thrombotic complications remain frequent despite pharmacologic thromboprophylaxis, suggesting that the thrombotic profile observed in COVID-19 differs from that seen in traditional forms of venous thromboembolism [14].

An important finding of the present study is the strong association between D-dimer elevation and pulmonary embolism. D-dimer reflects fibrin turnover and thrombus formation and has consistently been identified as a prognostic biomarker in COVID-19. Importantly, previous studies have demonstrated that markedly elevated D-dimer levels may not only predict thrombotic events but also reflect the intensity of systemic inflammation and endothelial activation [15]. In our cohort, peak D-dimer levels were almost ten-fold higher in patients with PE compared with those without PE, and peak D-dimer remained an independent predictor of PE in multivariable analysis, even after adjustment for acquired in-hospital risk factors and markers of clinical severity. The consistency of the effect estimates across the primary and sensitivity models further argues against major distortion of the observed associations by collinearity. This finding suggests that D-dimer may function as a clinically useful predictor of pulmonary embolism rather than solely reflecting global inflammatory burden. At the same time, D-dimer is closely intertwined with disease severity and systemic thromboinflammation in COVID-19, as supported by prior studies showing its association with adverse outcomes and endothelial activation. Taken together, these data suggest that D-dimer should be regarded as a “hybrid” biomarker that integrates both thrombotic load (and thus specific risk of PE) and overall disease severity, and that markedly elevated or rapidly rising values, particularly in the presence of additional risk factors such as prolonged immobilization or previous VTE, should lower the threshold for CTPA and may justify intensified thromboprophylaxis and closer monitoring. Therefore, interpretation of D-dimer levels in COVID-19 should always consider both thrombotic risk and overall disease severity. Although our observational design does not allow a complete separation of the thrombotic and inflammatory components, the persistence of peak D-dimer as an independent predictor in the adjusted model supports its dual role as both a marker of overall disease severity and a practical tool for identifying hospitalized COVID-19 patients at the highest risk of PE. These findings are consistent with the concept of immunothrombosis, in which inflammatory pathways interact with coagulation mechanisms and contribute to intravascular thrombus formation during severe systemic infections [16].

Prolonged immobilization also emerged as an independent predictor of pulmonary embolism in our cohort. While immobilization represents a classical risk factor for venous thromboembolism, its interaction with SARS-CoV-2-induced endothelial injury may amplify thrombotic risk beyond that observed in non-COVID-19 hospitalized populations. Experimental and clinical evidence suggests that endothelial dysfunction, platelet activation, and complement-mediated microvascular injury cause a prothrombotic milieu in COVID-19, conceivably explaining the high incidence of distal pulmonary thrombosis observed in imaging studies [17].

Interestingly, traditional cardiovascular risk factors such as age, sex, and baseline comorbidities were not independently associated with pulmonary embolism in this cohort, suggesting that acute disease-related factors may play a more dominant role than chronic cardiovascular conditions in determining thrombotic risk during COVID-19 hospitalization. This observation corresponds with previous reports indicating that inflammatory burden and disease severity may outweigh traditional risk factors in predicting thrombotic complications in acute SARS-CoV-2 infection [18].

From a pathophysiological perspective, pulmonary embolism in COVID-19 likely represents a spectrum ranging from classical embolic disease to in situ pulmonary thrombosis driven by endothelial injury and localized inflammation. Autopsy studies have demonstrated extensive pulmonary microthrombosis and endothelial inflammation distinct from patterns seen in non-COVID-19 acute respiratory distress syndrome, further reinforcing the view that SARS-CoV-2 infection has important vascular and endothelial components beyond the respiratory manifestations of the disease [19]. The predominance of segmental and subsegmental thrombi observed in our cohort further supports this hypothesis.

The possible implications of these findings may extend beyond the acute phase of infection. Although the present study did not include post-discharge follow-up, emerging evidence from other cohorts suggests that SARS-CoV-2 infection can be associated with persistent endothelial dysfunction, chronic low-grade inflammation, and an increased risk of cardiovascular events months after recovery [20,21,22]. In this context, acute pulmonary embolism during hospitalization could potentially serve as a marker of more severe vascular injury. However, whether PE itself contributes causally to long-term cardiovascular sequelae in COVID-19 survivors remains to be established in prospective studies with adequate follow-up. The current findings should therefore be regarded as hypothesis-generating in relation to post-acute COVID-19 syndromes (Long COVID).

From a clinical standpoint, the identification of patients at increased risk for pulmonary embolism during hospitalization may have important consequences for management strategies. Unlike many early multicenter reports that primarily quantified PE incidence, this analysis highlights the potential role of dynamic D-dimer trajectories and immobilization status in supporting clinical suspicion of pulmonary embolism and informing decisions regarding CTPA referral in hospitalized COVID-19 patients. Early recognition of high-risk profiles could inform decisions regarding intensified thromboprophylaxis, closer monitoring, and extended anticoagulation after discharge. Recent randomized trials have suggested possible benefits of therapeutic-dose anticoagulation in selected hospitalized patients with elevated thrombotic risk, although optimal strategies persist an area of current investigation [23]. Furthermore, the association between acute thrombotic episodes and possible long-term cardiovascular sequelae accentuates the importance of structured follow-up in survivors of severe COVID-19. Another clinically relevant aspect of our findings relates to anticoagulation strategies. In this cohort, pharmacologic thromboprophylaxis was initiated early in most hospitalized patients, and the majority of individuals who subsequently developed PE were receiving prophylactic-dose anticoagulation at the time of diagnosis, with only a minority already on intermediate or therapeutic regimens. This pattern suggests that PE in COVID-19 frequently occurs despite standard prophylactic dosing and underscores the heightened thrombotic milieu associated with SARS-CoV-2 infection. At the same time, we observed no clear signal that higher pre-PE anticoagulation intensity was systematically associated with lower PE rates, although the study was not powered to detect small differences between dosing strategies. These observations highlight the ongoing challenge of balancing the need for more aggressive anticoagulation in high-risk patients against the competing risk of bleeding, and support current recommendations favoring individualized, risk-adapted anticoagulation rather than a uniform escalation to therapeutic doses in all hospitalized COVID-19 patients.

The therapeutic challenge consists of balancing the increased thromboembolic susceptibility associated with SARS-CoV-2 infection against the potential risk of bleeding complications. COVID-19 has been characterized by a complex hemostatic imbalance, with evidence supporting both hypercoagulability and hemorrhagic vulnerability, indicating the need for individualized anticoagulation strategies [24,25].

### 4.1. Study Limitations

Several limitations should be considered when interpreting the findings of this study.

First, the retrospective observational design necessarily limits causal inference and introduces possible risks of selection bias, information bias, and unmeasured confounding. Although consecutive patient inclusion and standardized organizational procedures reduce some sources of bias, residual confounding cannot be excluded.

Second, pulmonary embolism diagnosis was based on clinically indicated computed tomography pulmonary angiography rather than systematic screening. Consequently, detection bias may have occurred, as patients with more severe disease or higher clinical suspicion were more likely to undergo imaging. This limitation is common among observational studies of thrombotic complications during COVID-19 and may lead to underestimation or overestimation of true incidence [26].

Third, although the overall cohort size was moderate, the number of pulmonary embolism events was relatively limited, which may restrict statistical power for detecting weaker associations and increase the risk of type II error. A formal a priori sample size or power calculation was not performed because of the retrospective design, and therefore the study may be underpowered to detect modest effect sizes or to support extensive multivariable modeling. Nevertheless, the observed effect sizes for key predictors were substantial, supporting the robustness of the primary findings.

Fourth, the study was conducted at a single tertiary care center, which may limit external validity and transferability to other populations with different demographic characteristics, treatment protocols, or medical systems. Regional variations in thromboprophylaxis practices and SARS-CoV-2 variants may likewise influence thrombotic risk profiles.

Fifth, longitudinal follow-up after hospital discharge was not available. Consequently, the study cannot directly evaluate long-term thrombotic outcomes or confirm causal relationships between acute pulmonary embolism and subsequent cardiovascular sequelae linked to long COVID. The discussion of long-term effects therefore remains hypothesis-generating.

Sixth, biomarker measurements were obtained as part of routine clinical care rather than standardized research protocols. Variability in measurement timing, frequency, and laboratory methods may introduce measurement bias. However, peak values were used when available to reduce this limitation.

Seventh, anticoagulation strategies evolved during the pandemic, mirroring changes in clinical guidelines and emerging evidence. Treatment heterogeneity may therefore represent an additional confounding factor changing both thrombotic risk and outcomes.

Finally, although multivariable models were constructed with attention to multicollinearity and model calibration, the possibility of model overfitting cannot be entirely excluded given the number of candidate predictors relative to the number of events. Future studies with larger cohorts and prospective designs are needed to validate the predictive associations identified in this analysis.

In our cohort, only 120 of 395 hospitalized patients (30.4%) underwent CTPA based on clinical suspicion of PE, and the multivariable prediction model was restricted to this imaged subset. As a result, the identified predictors reflect associations within a highly selected group enriched for patients with more severe or atypical presentations, rather than the entire hospitalized COVID-19 population, which introduces spectrum bias. Moreover, CTPA was used as the reference standard only in patients with clinical suspicion, so outcome verification was incomplete in the remaining ward population, which may lead to verification (referral) bias and affects the generalizability of the model coefficients. Therefore, the reported predictors and model performance should be interpreted as applying to patients with clinically suspected PE who undergo CTPA, not to all hospitalized COVID-19 patients.

Another important limitation relates to the relatively small number of PE events (*n* = 47) available for multivariable modelling in the CTPA subcohort. This results in a borderline events-per-variable ratio, which may increase the risk of overfitting and imprecise estimates of the odds ratios and model performance. We therefore restricted the final model to four clinically justified predictors and avoided additional covariates, but we acknowledge that penalized regression or further model simplification could potentially yield more stable estimates and should be considered in future external validations.

Missing data were handled using a complete-case approach, which reduces sample size and may introduce bias if the missing-at-random assumption is not met. Although the overall proportion of missingness for variables included in the multivariable model was low (all < 5%), this assumption cannot be formally verified and the resulting estimates should be interpreted with appropriate caution.

A further limitation is that the analysis of anticoagulation intensity was descriptive only, without formal statistical comparison or adjustment for confounding. In this observational setting, anticoagulation strategies were closely linked to disease severity and dynamic clinical status, so standard regression adjustment would only partially address confounding by indication and could yield misleading estimates. We therefore chose to present these data primarily to illustrate real-world patterns of anticoagulation rather than to infer causal effects on PE risk or clinical outcomes, and our findings should be interpreted as exploratory and hypothesis-generating. Future studies with larger event numbers and more detailed longitudinal data would be better suited to propensity score or marginal structural modelling approaches to more robustly account for confounding by indication.

### 4.2. Consequences for Risk Stratification

The identification of strong and clinically accessible predictors of pulmonary embolism has important consequences for risk stratification in hospitalized patients with COVID-19, particularly given the nearly ten-fold higher D-dimer levels observed in affected patients. The markedly elevated D-dimer levels observed in patients with pulmonary embolism support the role of this biomarker as a key screening tool for thrombotic complications. In clinical practice, markedly increased D-dimer concentrations, particularly when combined with acquired risk factors such as prolonged immobilization or prior venous thromboembolism, may help identify patients who warrant early diagnostic imaging, intensified thromboprophylaxis, or closer clinical monitoring.

These data also suggest that thrombotic risk in COVID-19 represents a dynamic process affected by both baseline vulnerability and in-hospital factors, stressing the importance of repeated clinical assessment rather than reliance on admission parameters alone. Early identification of high-risk profiles could promote personalized anticoagulation strategies and potentially reduce the burden of thromboembolic complications. Moreover, in light of emerging evidence from other studies linking acute thrombotic events with potential long-term cardiovascular sequelae, patients who develop pulmonary embolism during hospitalization might benefit from structured post-discharge cardiovascular follow-up. Prospective studies are needed to confirm this hypothesis.

### 4.3. Upcoming Directions and Research Priorities

In spite of these limitations, the present findings add to the growing body of evidence supporting the central role of thromboinflammation and endothelial dysfunction in COVID-19-associated cardiovascular complications. Identification of high-risk patients during hospitalization may promote personalized thromboprophylaxis strategies and inform post-discharge monitoring aimed at reducing long-term cardiovascular risk, which will need to be tested in prospective studies with dedicated follow-up.

Future research needs to focus on prospective multicenter studies with longitudinal follow-up to clarify the relationship between acute thrombotic complications and long-term cardiovascular outcomes, as well as randomized trials evaluating optimal anticoagulation strategies among different risk profiles. Importantly, prospective studies with systematic post-discharge follow-up are required to clarify whether acute PE during COVID-19 hospitalization is independently associated with increased long-term cardiovascular risk or long-COVID-related vascular complications. Integration of vascular imaging, endothelial biomarkers, and functional cardiovascular assessments may further improve understanding of post-COVID-19 cardiovascular pathophysiology.

## 5. Conclusions

Pulmonary embolism was identified in approximately one in eight hospitalized COVID-19 patients, highlighting a substantial thrombotic burden associated with SARS-CoV-2 infection and emphasizing the need for vigilant in-hospital risk assessment. In this cohort, markedly elevated peak D-dimer levels and prolonged immobilization emerged as the most powerful and clinically accessible independent predictors of pulmonary embolism, stressing the central role of acquired thrombotic risk factors during hospitalization. Patients with pulmonary embolism experienced more severe clinical courses, with higher rates of intensive care unit admission and need for advanced therapies, indicating that PE functions as a key marker of systemic thromboinflammatory activation rather than an isolated complication. The close interplay between immunothrombosis-related biomarkers, endothelial dysfunction, and pulmonary embolism further supports the concept of COVID-19 as a predominantly vascular disease, with potential contributions of acute thrombotic injury to long-term cardiovascular vulnerability and long COVID sequelae. Collectively, these findings advocate for dynamic D-dimer monitoring, careful evaluation of immobilization status, and personalized anticoagulation strategies to refine risk stratification, guide decisions regarding diagnostic imaging intensity, and inform structured post-discharge cardiovascular follow-up in hospitalized COVID-19 patients. Whether these patients carry an increased long-term cardiovascular risk attributable to the acute PE episode remains to be determined in future longitudinal studies. Beyond confirming previously described associations between D-dimer, immobilization, and PE, this study characterizes how these predictors perform in a contemporary, late-pandemic Eastern European cohort and supports their potential role in improving PE risk stratification in similar clinical settings. 

## Figures and Tables

**Table 1:** Baseline Characteristics of Patients According to Pulmonary Embolism Status.

Variable	No Pulmonary Embolism (*n* = 348)	Pulmonary Embolism (*n* = 47)	*p*-Value
Age, years	72.1 ± 13.6	68.7 ± 13.4	0.125
Male sex, *n* (%)	189 (54.3%)	24 (51.1%)	0.756
Body mass index, kg/m^2^	27.95 ± 5.11	28.37 ± 5.83	0.642
Current or former smoker, *n* (%)	115 (33.0%)	19 (40.4%)	0.328
Ischemic heart disease, *n* (%)	69 (19.8%)	6 (12.8%)	0.322
Prior venous thromboembolism, *n* (%)	29 (8.3%)	9 (19.1%)	0.031
Prolonged immobilization, *n* (%)	80 (23.0%)	29 (61.7%)	<0.001
ICU admission, *n* (%)	15 (4.3%)	6 (12.8%)	0.028
Length of hospital stay, days	12.2 ± 8.1	12.9 ± 8.6	0.594

Abbreviations: ICU, intensive care unit.

**Table 2:** Laboratory Parameters According to Pulmonary Embolism Status. Values are presented as median (interquartile range, IQR).

Parameter	No Pulmonary Embolism (*n* = 348)	Pulmonary Embolism (*n* = 47)	*p*-Value
D-dimer (peak), µg/L	529.5 (280–1320)	5322 (2140–10,000)	<0.001
Fibrinogen, mg/dL	462 (380–560)	498 (410–610)	0.072
C-reactive protein at admission, mg/L	108.0 (62–168)	113.8 (74–182)	0.318
Interleukin-6 at admission, pg/mL	38.5 (18–74)	49.2 (24–96)	0.091
Leukocyte count, ×10^3^/µL	7.8 (5.9–10.6)	8.6 (6.3–11.4)	0.146
Platelet count, ×10^3^/µL	219 (168–286)	231 (176–298)	0.284
Creatinine, mg/dL	1.02 (0.82–1.36)	1.08 (0.86–1.42)	0.411
Urea, mg/dL	46 (33–69)	49 (35–72)	0.537
INR	1.09 (1.01–1.18)	1.12 (1.03–1.21)	0.228
aPTT, seconds	29.4 (26.8–32.7)	30.1 (27.4–33.5)	0.301

Abbreviations: INR, international normalized ratio; aPTT, activated partial thromboplastin time. Values are presented as median (interquartile range, IQR).

**Table 3:** Characteristics of Pulmonary Embolism in Hospitalized COVID-19 Patients (*n* = 47).

Variable	Value
Pulmonary embolism localization	
Central (main or lobar arteries), *n* (%)	11 (23.4%)
Segmental, *n* (%)	24 (51.1%)
Subsegmental, *n* (%)	12 (25.5%)
ICU transfer related to PE, *n* (%)	6 (12.8%)
Thrombolytic therapy administered, *n* (%)	4 (8.5%)
PE-related mortality, *n* (%)	3 (6.4%)
Therapeutic anticoagulation, *n* (%)	47 (100%)
Duration of anticoagulation during hospitalization, days	10.2 ± 6.4

Values are presented as number (percentage) or mean ± standard deviation.

**Table 4:** Multivariable logistic regression model for predictors of pulmonary embolism in the CTPA subcohort (*n* = 120). The model yielded an AUC of 0.80 for predicting PE.

Variable	Adjusted OR	95% CI	*p*-Value
Peak D-dimer (log-transformed)	3.9	2.1–7.1	<0.001
Prolonged immobilization	5.1	2.4–10.9	<0.001
Prior venous thromboembolism	1.8	0.8–4.2	0.16
ICU admission	2.4	0.9–6.6	0.09

## Data Availability

De-identified clinical, laboratory, and imaging data supporting the findings of this study are available from the corresponding authors upon reasonable request, subject to institutional data-sharing policies.

## Abbreviations

The following abbreviations are used in this manuscript:

aPTT	activated partial thromboplastin time
AUC	area under the curve
BMI	body mass index
CI	confidence interval
COVID-19	coronavirus disease 2019
CTPA	computed tomography pulmonary angiography
ICU	intensive care unit
IL-6	interleukin-6
IQR	interquartile range
OR	odds ratio
PASC	post-acute sequelae of COVID-19
PE	pulmonary embolism
ROC	receiver operating characteristic
RT-PCR	reverse transcription polymerase chain reaction
SD	standard deviation
STROBE	Strengthening the Reporting of Observational Studies in Epidemiology
VIF	variance inflation factor
VTE	venous thromboembolism
